# Stack or trash? Quality assessment of virtual slides

**DOI:** 10.1186/1746-1596-8-S1-S23

**Published:** 2013-09-30

**Authors:** David Ameisen, Christophe Deroulers, Valérie Perrier, Jean-Baptiste Yunès, Fatiha Bouhidel, Maxime Battistella, Luc Legrès, Anne Janin, Philippe Bertheau

**Affiliations:** 1 Institut Universitaire d'Hématologie, Université Paris-Diderot, F-75010 Paris, France; 2 Service de Pathologie, Hôpital Saint-Louis, APHP, F-75010 Paris, France; 3 Laboratoire de Pathologie, Inserm UMR_S728 / Université Paris-Diderot, F-75010 Paris, France; 4 IMNC - UMR 8165 CNRS / Université Paris-Diderot, Université Paris-Sud, F-91405 Orsay, France; 5 Laboratoire Jean-Kunztmann, Université de Grenoble / CNRS, UMR 5224, 38041 Grenoble Cedex 9, France; 6 Laboratoire LIAFA, Université Paris Diderot, Sorbonne Paris Cité, 75205 Paris Cedex 13, France

## Background

Since microscopic slides can now be automatically digitized and integrated in the clinical workflow, quality assessment of these Whole Slide Images (WSI) has become a crucial issue. At this time, the quality of a WSI is verified a posteriori by a technician or by a pathologist. There is however a significant amount of WSI that are too insufficient in quality (blurred, bad colors, poor contrast.) to be used for diagnoses. These slides have then to be scanned again with delay thus slowing down the diagnostic workflow.

To address this problem, we chose to design a method of quality assessment followed by reacquisition, as opposed to a process of enhancement or restoration [1,2]. Such process indeed too frequently results in the degradation of image quality, a key factor in medical diagnosis.

The quality of a flat image can be defined by several quantifiable parameters such as color, brightness, and contrast. One of the most important parameters, yet difficult to assess, is the focus sharpness (i.e. the level of focus blur) [3]).

Quality assessment of WSI is much more complex than that of flat images because of their intrinsic structure made of multiple magnification levels (pyramidal structure) and resolutions above the gigapixel. One study [4] has shown the possibility of comparing the tiles’ contrast and entropy in two WSI obtained with two different scanners digitizing the same slide. Another work [5] assessed the focus sharpness of the tiles of a WSI with the generation of a focus assessment map of the WSI at a given magnification level. However, both these methods still require a human eye to assess if the WSI must be accepted or discarded after the scan.

We describe here a fast method to automatically assess quality and to accept or discard WSI at the time of acquisition.

## Material and methods

### Material and software

For the computations that follow, we used a machine at the University Paris Diderot

Paris 7, with the following configuration: 2 Quad-Core Xeon X5450 3.0GHz/2x6MB, 8GB 667MHz FBD RAM.

The program implementing the new quality assessment method has been developed in Java Web with the NetBeans 6.1 Integrated Development Environment, the Tomcat 6 application server and the database server MySQL 5.

The web survey was developed in PHP5 and MySQL 5.

The tiles of each magnification level of the WSI need to be accessible to perform the analysis. Many open-source programs [6,7] as well as proprietary ones [8] can be used to extract WSI files from different formats (3dHistech, Aperio, Hamamatsu, Olympus) into series of tiles at different magnification levels.

### Methods

Once the tiles are extracted, the saturation of each of them is computed. In every system, many “blank tiles” are stored because they contain visual artifacts detected as regions of interest but do not contain any specimen. As these blank tiles have saturation values close to zero, our system discards them from the set of images to analyze, saving from 5 to 40 percents of the time required to complete an analysis of a virtual slide at maximum magnification.

The remaining tiles are then analyzed with different tests such as blurriness, contrast, brightness and color. More tests can be integrated as plug-ins in the program. For the blurriness assessment we used a fast reference-free method designed to compute accurately the amount of blur in a single tile based on an edge brightness ratio [9]. Other tests such as contrast, brightness and color assessment are a result of computations made on the tile’s pixels vales, compared with their respective thresholds. For instance, one test could be to check if more than 90% of the pixels color values inside a tile were contained in three ranges of color.

Each tile receives quantitative and qualitative scores for each of the analyzed parameters and are compared to their respective thresholds. Note that the tiles can be virtually split to add granularity and refine the final assessment. For instance, at a 2x magnification, if more than 90% of the tiles are considered sharp, the complete 2x layer of the WSI is considered as sharp. If more than 70% of the 10x magnification is considered sharp, the 10x layer of the WSI is considered as sharp.

The analysis can be limited to the lower magnification levels of a WSI for a quicker result or extended to the highest magnification level for a more comprehensive quality assessment.

Once the tile analysis is done, if the WSI passed the quality assessment tests at each processed layer of magnification the WSI is suitable for further use.

In order to test and validate the method, we analyzed a series of 100 WSI made of a mix of WSI with optimal focus and of WSI with various blurred areas, some of them being obviously totally blurred. We compared the computer assessment of these WSI to the human assessment in two settings:

- We first presented the 100 WSI in a random order to two observers from our research team.

- We then conducted a web survey [10] among 22 trained pathologists, asking them whether the overall quality of each WSI seemed sufficient for a clinical use. The human assessment was distributed among three possible answers: Poor; Fair; Good. The computer assessment represented the computed highest acceptable magnification for a WSI, higher magnifications being therefore considered by the computer as of insufficient quality for diagnosis.

## Results and discussion

In the following, we use the blur assessment method described in the method section as an example to describe any other quantifiable criterion in an image, to be used a fortiori to assess the quality of WSI.

The complete quality assessment method is a logical intersection of independent tests, marking a WSI as of insufficient quality if at least one of the tests fails.

**Figure 1:**
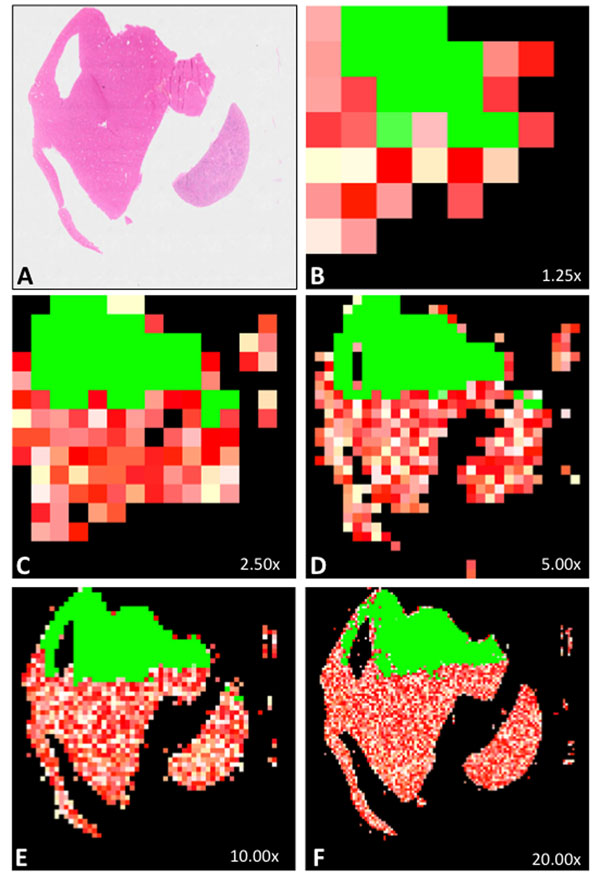
**Automatic quality analysis of a virtual slide (parameter used: blur) ***A* represents the thumbnail of a whole slide image (H&E staining) whose upper third part is in focus and lower two thirds part is totally out of focus. Each thumbnail *B* to *F* shows sharp tiles in green and blurry tiles going from white (a little blurry) to red (the most blurry). Out of 43 tiles at 1.25x (*B*), 83% were detected as non-blank, and 36% were detected as sharp. For *C*, *D*, *E* and *F*, the respective values were (146 tiles, 2.5x, 86% non-blank, 34% sharp), (493 tiles, 5.0x, 83% non-blank, 33% sharp), (1751 tiles, 10.0x, 77% non-blank, 31% sharp), (6589 tiles, 20.0x, 76% non-blank, 25% sharp). The WSI is thus considered as of insufficient quality in terms of blurriness, for all its magnification levels being under their respective blur assessment thresholds.

We applied the quality analysis routine with the blur assessment parameter on hundreds of WSI. An example of automatic blur assessment is shown in Figure 1.

On a collection of 100 WSI, two observers could easily assess the overall level of quality they observed and they visually verified that the thresholds we set were highly predictive of the global sharpness or blurriness of the WSI.

**Figure 2:**
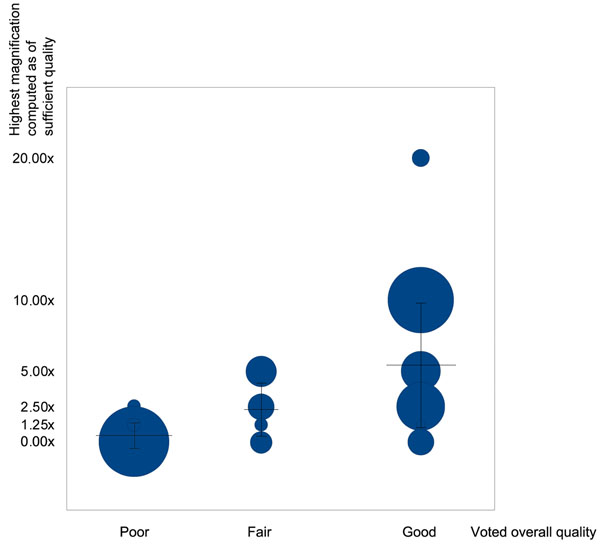
**Comparison between voted overall quality and best detected magnification** Distribution of the (human assessment; computer assessment) pairs for 100 WSI with various blurred levels. Human assessment is distributed in three categories: poor/fair/good quality for diagnosis. Computer assessment is distributed in five different magnifications (from 1.25X to 20X): it shows the highest acceptable magnification for a WSI, i.e. the magnification for which the WSI computed quality is sufficient, implying that higher magnifications of this WSI are of insufficient quality. The surface of the disk is proportional to the number of identical pairs. The horizontal bars represent the mean of the highest acceptable magnifications of the computer assessment at each category of human assessment, with vertical bars as their respective standard deviation.

For the web survey, the results [10] obtained after the visual analysis on 100 WSI by 22 pathologists are shown in Figure 2. The results found by our algorithms are fully consistent with the pathologists’ answers to the survey: the mean computer assessment is 1.25X with a standard deviation of 2.37X in the “poor” human assessment category, increasing to 2.90X with a standard deviation of 2.51X in the “fair” category and to 6.35X with a standard deviation of 5.57X in the “good” category.

However, the survey showed that the human assessment do not entirely correspond to the computer assessment, due to the fact that some diagnoses do not need high magnification for human eyes to be done. Indeed, a high computer quality at low magnification was sometimes enough to give a correct diagnosis (blue disks on the lower right part of Figure 2), but a high-level computer assessment (computed high quality at high magnification) always corresponded to a high level human assessment (blue disks on the upper right part of Figure 2).

As further improvements of our method, we will contextualize the assessment by refining the thresholds depending on staining and lesion.

In terms of computing speed, Zerbe *et al*.[5] showed a distributed computing model to assess the focus sharpness of a WSI, generating a focus assessment map of the WSI at a given magnification level in around 6 minutes per gigapixel per computer. We analyzed on our machine (see Material and software sub-section) 8 complete 1.73 gigapixel digital slides in 400 seconds as eight distinct threads, equivalent to 34 Megapixels per second or 2 gigapixels per minute, per computer. Already 12 times faster than the previous method, we are currently optimizing the program into a multi-thread, multi-node parallel processing system using C++ with OpenMP and OpenMPI libraries to scale it up to match demanding industry requirements. A plug-in support and an API are also being integrated in this optimization to facilitate further integration.

## Conclusions

As quality assurance is crucial in a context of daily use in diagnostic pathology, we have developed a fast and reliable reference-free tool for quality assessment of WSI.

Our method can be used upstream, as a calibration and quality control tool for the WSI acquisition systems, or as a tool to reacquire tiles while the WSI is being scanned. It can also be used downstream to reacquire the complete slides that are below the quality threshold for surgical pathology analysis.

We are currently optimizing the program to improve its speed and refining its threshold, according to the magnification levels, the staining of the slides, and the type of acquisition devices used.

Such quality assessment scores could be integrated as metadata in WSI shared in clinical, research or teaching contexts, for a more efficient medical informatics workflow.

## Competing interests

### Financial competing interests

David Ameisen was a recipient of a doctoral fellowship grant from Aurora Interactive (2008-2011); Olympus provided him travel reimbursements for scientific meeting presentations. None of these organizations are financing this manuscript. No other author has financial competing interests.

David Ameisen and Philippe Bertheau are currently applying for two patents relating to the content of this manuscript. They are receiving salaries from Université Paris Diderot that has applied for these patents.

### Non-financial competing interests

None

## Authors' contributions

DA participated in the design of the study and drafted the manuscript, CD carried out the Hamamatsu tiles extraction and participated in the design of the study, VP participated in the design of the study, JBY participated in the design of the study, FB participated in the statistical analysis, MB participated in the statistical analysis, LL participated in the statistical analysis, AJ participated in the design of the study, PB participated in the design of the study, and drafted the manuscript. All authors read and approved the final manuscript.
